# Supplementary figures and images for: Distance and grid-like codes support the navigation of abstract social space in the human brain
Source: eLife. 2024 Jun 14;12:RP89025. doi: 10.7554/eLife.89025 (PMC11178359; doi:10.7554/eLife.89025)

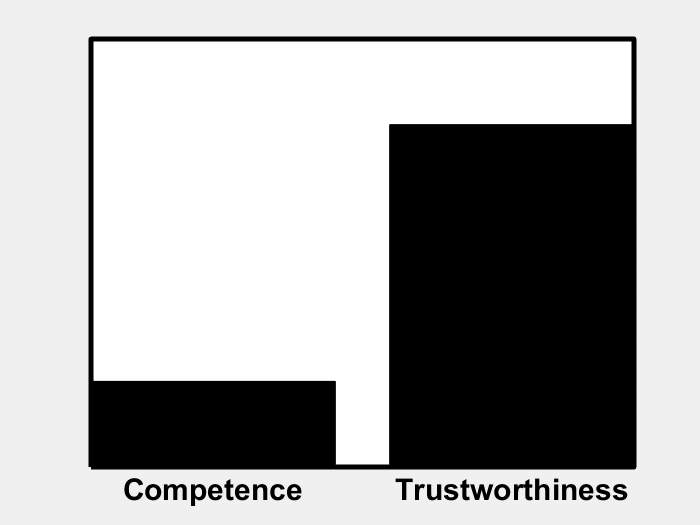

Supplement: Supplementary file 1 [file elife-89025-fig1-video1.gif]
